# Supplementary material for: Coherent microwave comb generation via the Josephson effect
Source: Nat Commun. 2026 Feb 20;17:2972. doi: 10.1038/s41467-026-69652-1 (PMC13036065; doi:10.1038/s41467-026-69652-1)
Supplement: Supplementary file 1 — Supplementary Information [file 41467_2026_69652_MOESM1_ESM.pdf]

Coherent microwave comb generation via the Josephson effect: Supplementary Information

SUPPLEMENTARY NOTE 1: $\phi_{\mathbf{dc}}$ CALIBRATION BY REFLECTOMETRY

By measuring the phase change of a probe signal reflected by the dc SQUID, one can quantify the flux induced in the loop with respect to the control parameter at room temperature, typically the bias current. The blue dots in Supplementary Fig. 1 show the phase shift of a probe tone at 4.072 GHz impinging on the SQUID as a function of the applied flux Φ_{dc}. The dashed line represents the fitted theoretical curve to extract some parameters of the device. The room temperature tunnel resistance of the SQUID is $\sim 1.7\,\mathrm{k\Omega}$, hence by considering an increase of 10% in the normal state resistance R_n at cryogenic temperature we estimate a total tunnel resistance of $1870\,\Omega$ at operating conditions. By considering the superconducting gap of Al devices usually obtained in our lab, and by using the Ambegaokar-Baratoff formula we estimate the critical current to be $I_c = 144\,\mathrm{nA}$. I_c is kept fixed and the fit runs with two free parameters: the SQUID intrinsic capacitance C and the asymmetry coefficient defined as

$$r = \frac{I_{c1} - I_{c2}}{I_{c1} + I_{c2}}. \tag{S1}$$

The phase rotation of a microwave tone at the termination of a waveguide can be expressed as [1]

$$\theta = \tan^{-1}\left(\frac{2\sin(\arg Z)}{\left(\frac{|Z|}{Z_0} - \frac{Z_0}{|Z|}\right)}\right), \tag{S2}$$

where Z_0 is the characteristic impedance of the line and Z is the input impedance of the SQUID. For Z we consider the Resistively and Capacitively Shunted Junction (RCSJ) model neglecting the resistive branch, hence by evaluating the parallel of the inductive plus capacitive branches. It turns out that $Z = Z_C // Z_L$, with $Z_C = 1/j\omega C$ and $Z_L = j\omega L_J$, with L_J the Josephson inductance. It is important to underline that while sweeping the magnetic flux the superconducting phase across the SQUID has to be changed according to $\varphi = \arctan(-r\tan\phi)$, since $L_J = L_J(\varphi)$. The best fit yields $C = 40\,\mathrm{fF}$ and $r = 0.05$. By construction the loop inductance is $L = 100\,\mathrm{pH}$, which gives a screening factor $\beta_L = 0.014$. As a consequence, the screening effect is not taken into account in the fit.

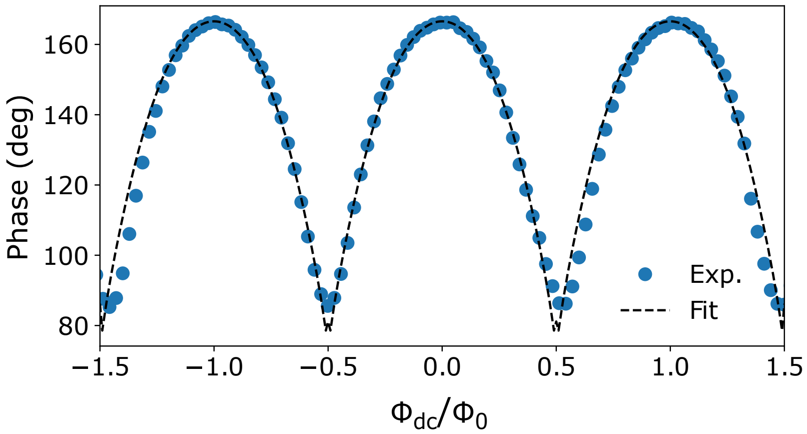

Supplementary Figure 1. Reflectometry data and fit with model. The evolution of the phase of the S21 parameter shows the flux modulation of the dc SQUID, which allows the calibration of the flux bias. The Vector Network Analyzer is set in continuous wave mode with an applied tone of frequency 4.072 GHz.

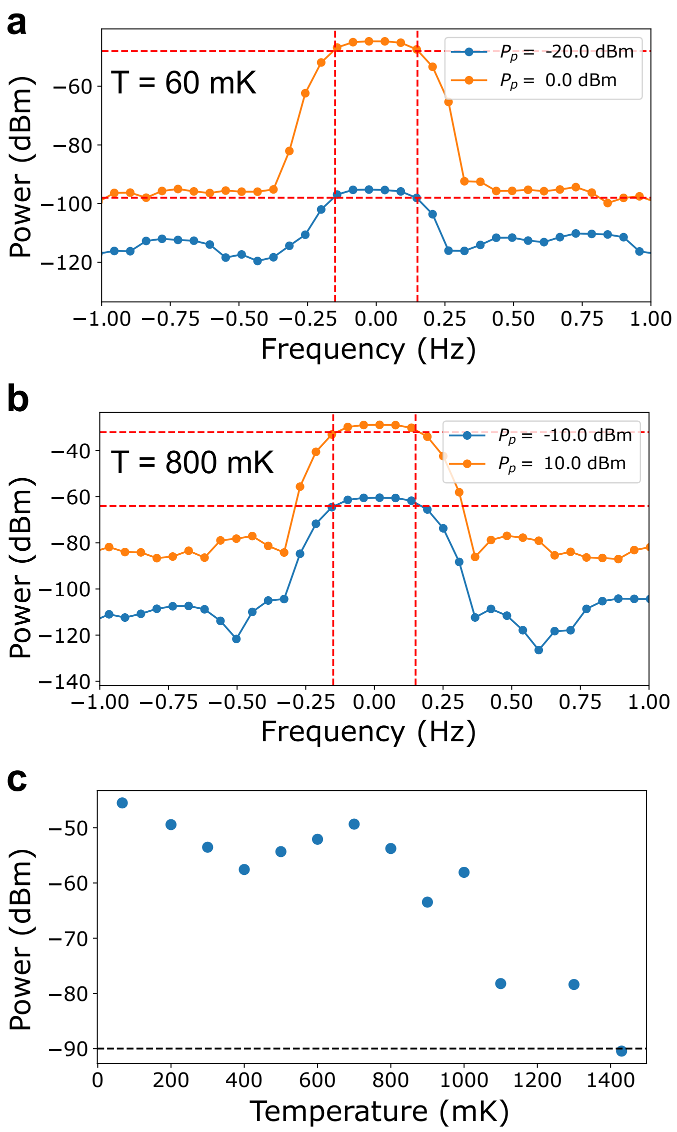

Supplementary Figure 2. Spectrum measured at 60 mK (**a**) and 800 mK (**b**) showing the 5th harmonic of a frequency comb generated using a pump frequency of 833.34 MHz with two different pump powers. The horizontal lines show the -3 dB point of the two peaks, and the vertical lines the corresponding linewidths. **c** Intensity of the 7th harmonic of 833.34 MHz as a function of the bath temperature. The black dashed line indicates the noise floor of the raw spectra.

SUPPLEMENTARY NOTE 2: TEMPERATURE DEPENDENCE OF COMB MODES

In this section, we compare the linewidth of a comb mode generated at base temperature $T_1 = 60\,\text{mK}$ and at higher temperature, $T_2 = 800\,\text{mK}$, about $0.7T_c$, with T_c the critical temperature of the aluminum ($\approx 1.25\,\text{K}$ in our case). During the measurements, $\Phi_{\text{dc}} = \Phi_0/2$. Supplementary Fig. 2 shows the 5th harmonic of a frequency comb generated using a pump frequency of 833.34 MHz at T_1 and T_2, in panel a and b respectively, for two different pump powers. In the two plots the $-3\,\text{dB}$ points, indicated by the horizontal lines, coincide with a full width at half maximum of 0.3 Hz, which again is the lower bound limitation given by our spectrum analyzer. Moreover, the plots do not show any significant dependency on the pump power. In panel c, the 7th harmonic amplitude does not display a monotonic dependence on bath temperature and remains clearly detectable until T_c is reached.

SUPPLEMENTARY NOTE 3: SPICE SIMULATIONS OF COMB EMISSION

Comb modes as a function of Φ_{dc} and comparison with experiment

Numerical simulations are performed by using JoSIM, a SPICE-based circuit solver developed by Johannes Delport [2]. The physical behavior of the system is well captured by the simple circuit shown in Supplementary Fig. 3. The

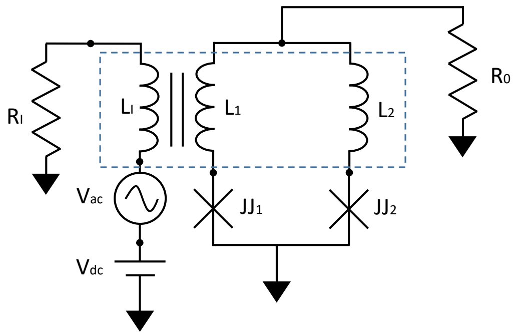

Supplementary Figure 3. Circuit used to perform SPICE simulations.

diagram is composed by a flux line (left), powered by a dc voltage source V_{dc} with an ac voltage source V_{ac} in series. The flux line is terminated by a resistor R_I which turns the applied voltage into current, and presents an inductive coupling between L_I, L_1 and L_2. The former is the inductance of the pump line, the latter two represent the self-inductance of the SQUID, and are designed with a coupling constant respectively of -0.5 and 0.5 with respect to L_I, so that the induced phase drop across them is coherent with respect to the circulating current. The Josephson junctions embedded in the SQUID (right) are represented by the elements JJ_1 and JJ_2, while a resistor R_0 plays the role of a matched load. The parameters used in the simulations are set to match the characteristic of our SQUID. In particular, $L_1 + L_2 = L_{loop} = 100\,\mathrm{pH}$ by construction. The parameters of the Josephson junctions are chosen in agreement with the fit of the reflectometry data in Supplementary Fig. 1. R_I is tuned to the value of the dc resistance of the flux line, and in our case goes from $1\,\mathrm{k}\Omega$ to $10\,\mathrm{k}\Omega$ depending on the setup. The inductance L_I is chosen so that the mutual inductance $M = k\sqrt{L_{1,2}L_I}$ is equal to the value of Sample 1 and Sample 2 determined experimentally.
The model for the Josephson junction used in JoSIM is a standard RCSJ model. The shunt resistance is a piecewise function with two different values below and above the energy gap, that physically represent the subgap resistance R_{SG} and the normal state resistance R_N respectively. In the simulation they can be chosen separately depending on the type of junction. Such general definition of the shunt resistance is physically accurate, and allows to take into account the power dissipated when the device develops a finite voltage drop but keeps being superconductive [3].
The simulations are performed by varying the values of the voltage source V_{dc} to move Φ_{dc}, and V_{ac} to modify Φ_{ac}. JoSIM is a time-domain circuit solver, hence a time trace of the voltage drop across the load resistor R_0 is generated. We then perform a Fourier transform on the generated time series and record the amplitude of the harmonics as a function of V_{dc} and V_{ac}.
The one-dimensional traces in Fig. 2e of the main text are high-resolution scans of the flux offset to highlight the fine structures of the harmonic amplitudes function of Φ_{dc}. The plot shows one odd and one even harmonic, the 7th and the 8th, respectively. The experimental parameters of the power spectrum analyzer for data acquisition are: resolution bandwidth 10 Hz, span 1 kHz, 5 averages. About the drive tone, pump power is $-3\,\mathrm{dBm}$ and pump frequency 597.34 MHz.
As well reproduced by the simulations in Fig. 2f, both the harmonics have even parity with respect to $\Phi_0/2$. One can also notice that the number maxima presented by both curves is equal to the order of the harmonic, hence the 7th harmonic presents 7 maxima while the 8th has 8 maxima. It can be shown that this is a general property and holds for every harmonic of the comb. Finally, we notice that at $\Phi_{dc} = \Phi_0/2$ the odd harmonics have the highest maximum, while even harmonics have a minimum. This is due to the fact that at $\Phi_0/2$ the voltage pulses are equally spaced and with alternated signs, yielding a Fourier transform with only odd harmonics.

Simulations of voltage pulses

Here we report a section of the train of voltage pulses and the corresponding comb spectrum obtained from JoSIM simulations with the circuit and parameters just discussed.
Supplementary Figure 4a shows the first 10 harmonics of a pump tone at 1 GHz such that $\Phi_{ac} < \Phi_0$ and $\Phi_{dc} = 0.95 \times \Phi_0/2$. The odd harmonics are the most prominent ones, as the bias approaches half flux quantum, a node for the even harmonics (recall the paragraph above and Fig. 2e in the main text). Above the 10th harmonic the power of each mode is below -130 dBm, in agreement with our estimations of the parameters of the readout circuitry.
Supplementary Figures 4b and c display a sequence of voltage pulses with pump frequency of 1 GHz and 100 MHz, respectively. The time scale is chosen to show a time period, i.e. two pulses with alternated sign on voltage axis, of a pump tone at 100 MHz. In either plot, we estimate a voltage amplitude of each of few microvolts, and a width of few hundreds of picoseconds.

SUPPLEMENTARY NOTE 4: MEASUREMENT SETUP

The schematic of the measurement setup used in the experiment is shown in Supplementary Figure 5. The left part of the diagram reports the circuitry for the flux line, which is inductively coupled to the loop of the dc SQUID. The right part is the output line that starts from one of the SQUID and terminates at room temperature with the instruments for data acquisition.
The pump signal provided to the comb generator is a sinusoidal tone that is first attenuated and then summed at room temperature using a bias tee with a dc bias current generated by a low-noise dc source. The signal is then sent to the mixing chamber of the dilution fridge via low-loss coaxial lines with a cut-off frequency of ~ 1.5 GHz given by the combination of bandwidth of coax cables, multi-stage cryogenic filters and eccosorb filters (made by Karlsruhe Institute of Technology). The pump signal is routed similarly along the way out to room temperature, so that by input-output measurements we can quantify the frequency-dependent attenuation of the pump signal down to the sample. In the experiment, the way back is terminated by a 50 Ω cap put at the rf port of an other bias tee.
The comb signal generated by the SQUID is conveyed to the amplification chain by an on-chip CPW and then a rf-switch, followed by a triple-stage cryogenic isolator. Between the circulator and the HEMT amplifier, we put a high-pass filtering stage with cutoff frequency always higher than the pump tones. This ensures a strong suppression of the pump tone at the HEMT input port, avoiding the amplifier saturation and damaging. After a further amplification of the signal at room temperature, three types of measurements are performed: 1) time-stability of the comb modes by heterodyne down-conversion, 2) direct frequency spectroscopy of the emitted lines, and 3) reflectometry measurements as a function of Φ_{dc}.
Configuration 1) is indicated by the cyan wiring in Supplementary Figure 5. After room temperature amplification, an IQ mixer is used to shift the comb spectrum down to the lock-in bandwidth. The in-phase (I) and quadrature (Q) components are pass-band filtered and then acquired simultaneously by a digital high-frequency lock-in. To probe a single comb mode (Fig. 3 of main text), the two quadratures are demodulated to baseband by an internal oscillator of the lock-in. The resulting signal is integrated with a 1 ms time constant and then recorded at $\sim 14 \times 10^3$ samples/second for 10 seconds.
The histograms in Fig. 4b are acquired in a similar way. The phase of the pump tone is stepped eight times every 2 seconds, and the down-converted signal is recorded continuously at $\sim 27 \times 10^3$ samples/second once low-pass filtered with a 1 ms time constant. In Fig. 4d the digitization follows the same parameters, but now we address the phase of 5 harmonics by 5 synchronous demodulators.
The acquisition of the power spectra 2) is performed by directly sampling the spectra of the amplified signal with a spectrum analyzer (orange wire in Supplementary Fig. 5).
Finally, the reflectometry circuit 3) enables the flux calibration by Supplementary Fig. 1. The vector network analyzer applies a single-tone signal that is delivered to the SQUID by means of the last stage of the circulator. In this way, the phase of the back-reflected signal depends on the inductance of the load, which is the flux-modulated SQUID. The circulator separates the outgoing wave, which is then amplified and finally acquired by the vector network analyzer itself (green circuitry in Supplementary Figure 5).
To properly rescale the measured power shown in the plots to the power emitted at sample output, we have to quantify the amplification along the readout line. As argued in the main text, the overall gain of the output line from output port of the sample to instrumental acquisition ranges between 87 dB at 4 GHz and 82 dB at 8 GHz (indicated for brevity as 87-82 dB). Such numbers include $\sim$ 30-20 dB overall amplification in the same 4-8 GHz bandwidth across the output line of the cryostat (copper coaxial from sample to circulator, NbTi superconducting coaxial from

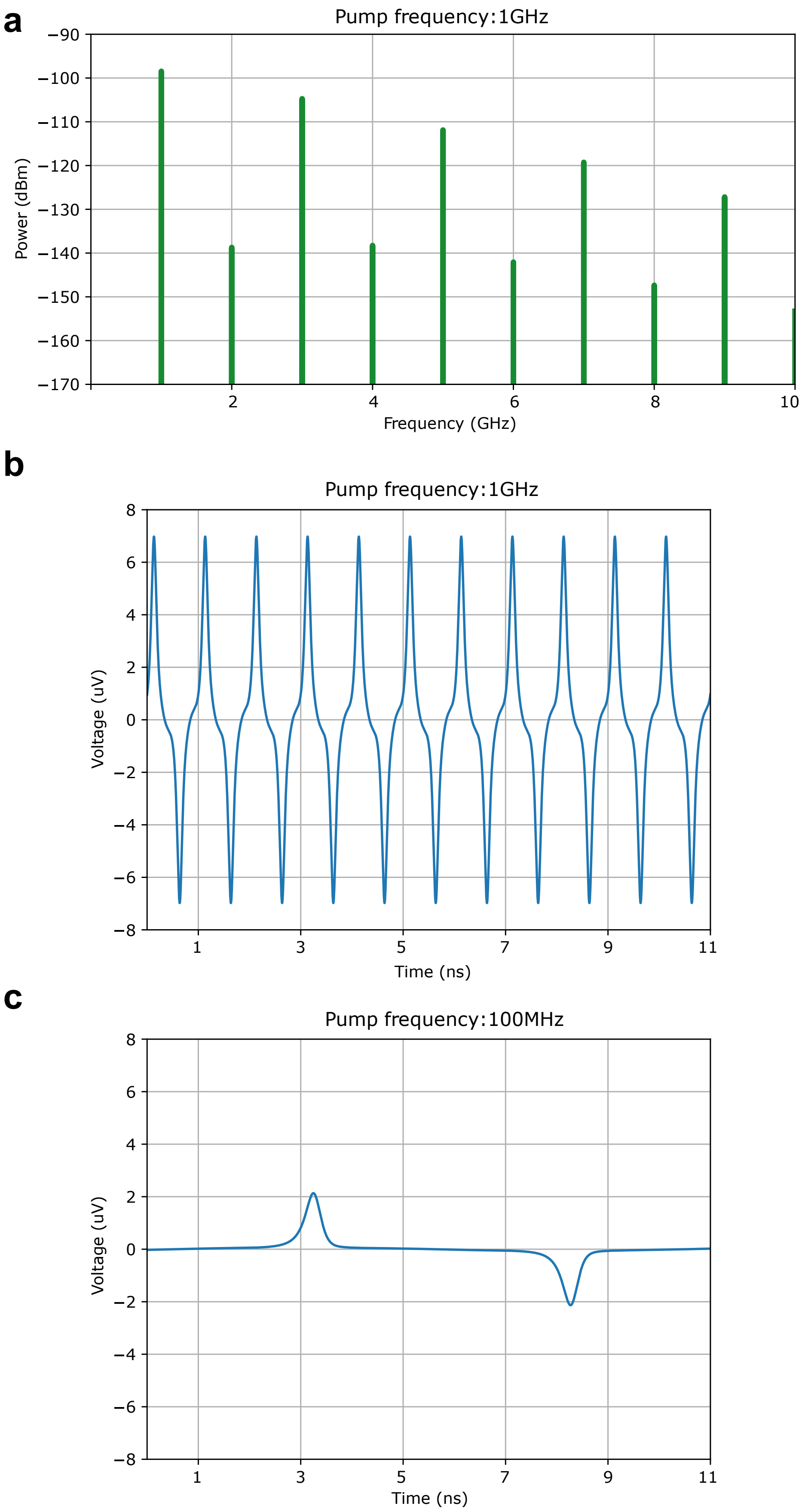

Supplementary Figure 4. Simulated comb spectrum (**a**) and train of voltage pulses emitted by the device (**b**) with a pump signal of 1 GHz. **c** Period of voltage pulses for a pump signal at 100 MHz.

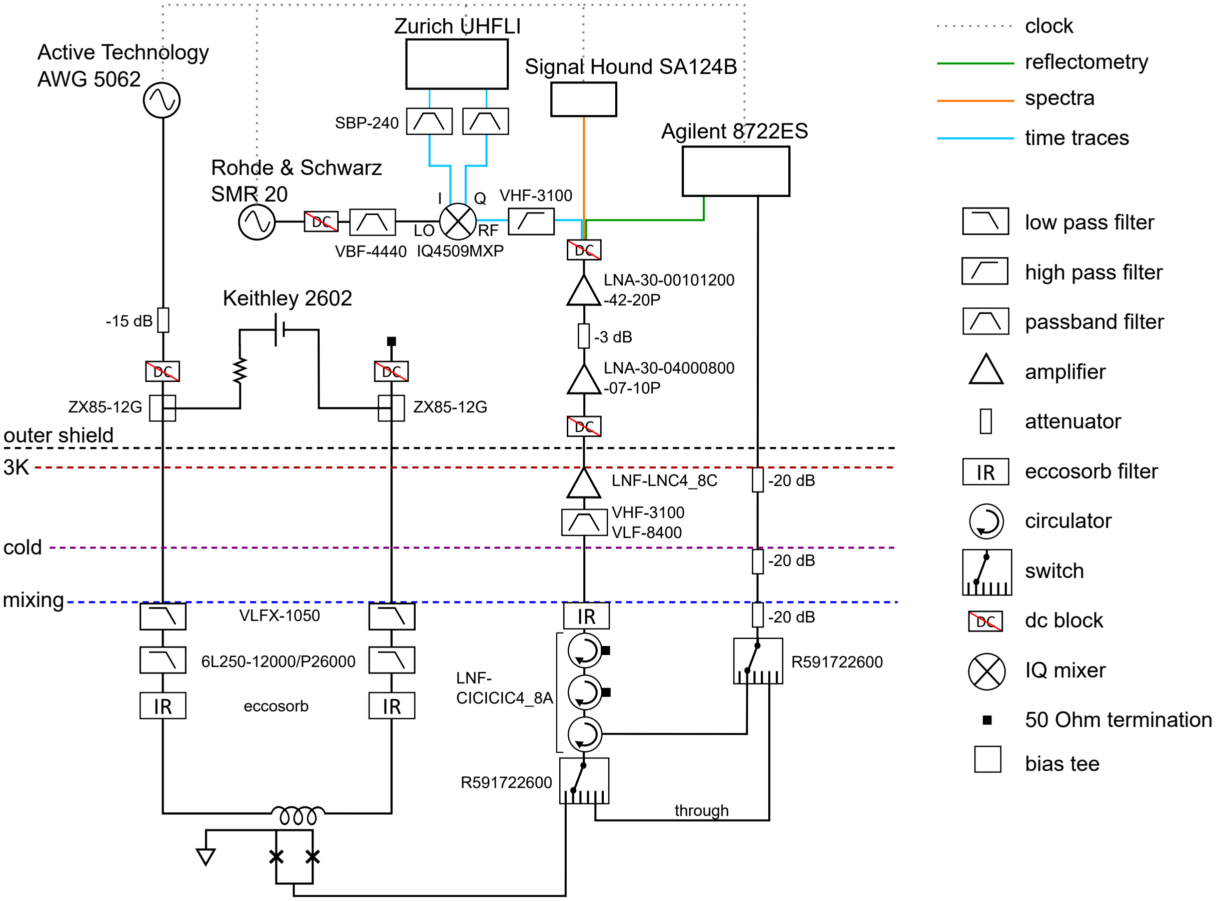

Supplementary Figure 5. Depending on the experiment, the amplified signal is analyzed differently. Each setup is color coded (cyan, orange, and green). The rf sources and measurement instruments share the same clock reference.

circulator to HEMT, HEMT, and CuNi coaxial cable from HEMT to output connector) and the further two-stage room temperature amplification ($+28\,\mathrm{dB} - 3\,\mathrm{dB} + 33\,\mathrm{dB}$).

SUPPLEMENTARY NOTE 5: NOISE IMPACT ON IQ DISTRIBUTIONS

The sizable width of the IQ distributions suggests the presence of broadening by thermal noise from the amplifier chain and other possible noise sources, as E_J fluctuations or pump instabilities.
To appreciate the impact of the detection circuit, we compare the IQ distribution of a single mode when the drive tone is switched on and off. In the ”pump off” condition, the generator emission spectrum is approximately zero, as having no resonator implies that there is neither cavity occupation due to thermal photons, nor a power spectral density emitted from a cavity. Therefore, the noise signal collected is mainly due the cold HEMT amplifier at the first stage of amplification.
Supplementary Figure 6a shows the IQ distribution of such signal (”pump off”), together with the data of the 9th harmonic of $f_p = 597\,\mathrm{MHz}$ (”pump on”). Both histograms are obtained with the same demodulation parameters of Fig. 3c and Fig. 4b. The two distributions are displaced from each other and are both nearly isotropic in the IQ plane.
In Supplementary Figure 6b, one-dimensional cuts and Gaussian fits show that the two distributions have comparable widths, a hallmark of amplitude fluctuations at the generator level below our sensitivity.

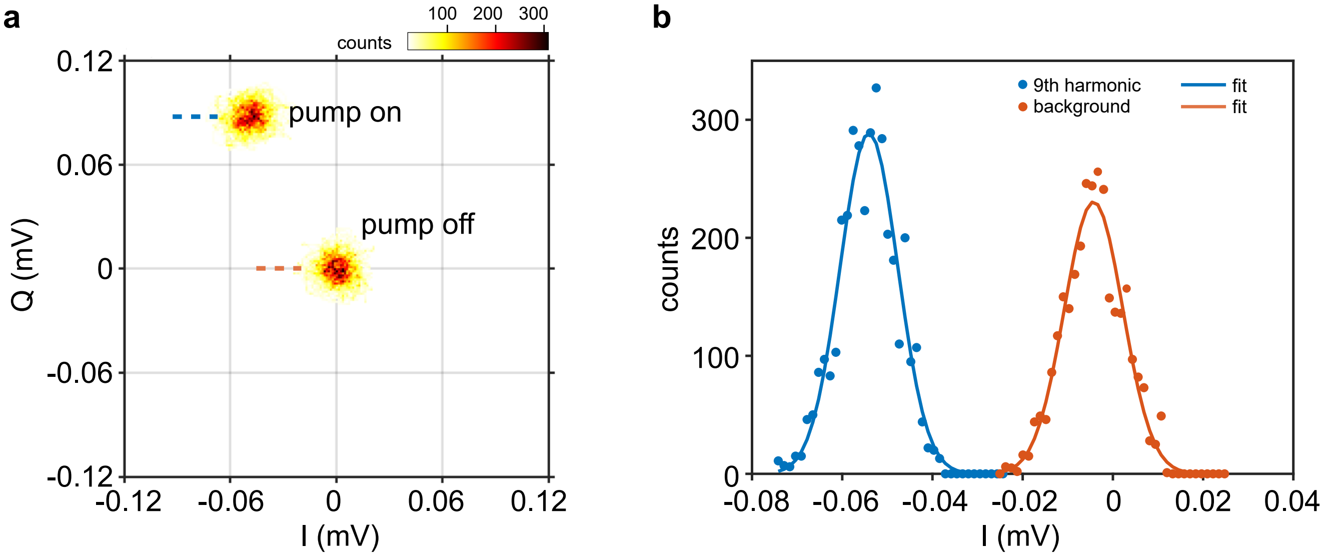

Supplementary Figure 6. **a** Histograms of the demodulated signal of the 9th harmonic of $f_p = 597\,\mathrm{MHz}$, with amplitude -5 dBm and phase 0 deg ("pump on", same dataset of Fig. 4b) and of the background signal due to the readout circuitry ("pump off"). **b** One-dimensional horizontal slices of the two distributions along the color-coded dashed lines in panel a. The Gaussian fits return similar widths: $(0.0064 \pm 0.0006)\,\mathrm{mV}$ for the 9th harmonic and $(0.0065 \pm 0.0005)\,\mathrm{mV}$ for the background signal.

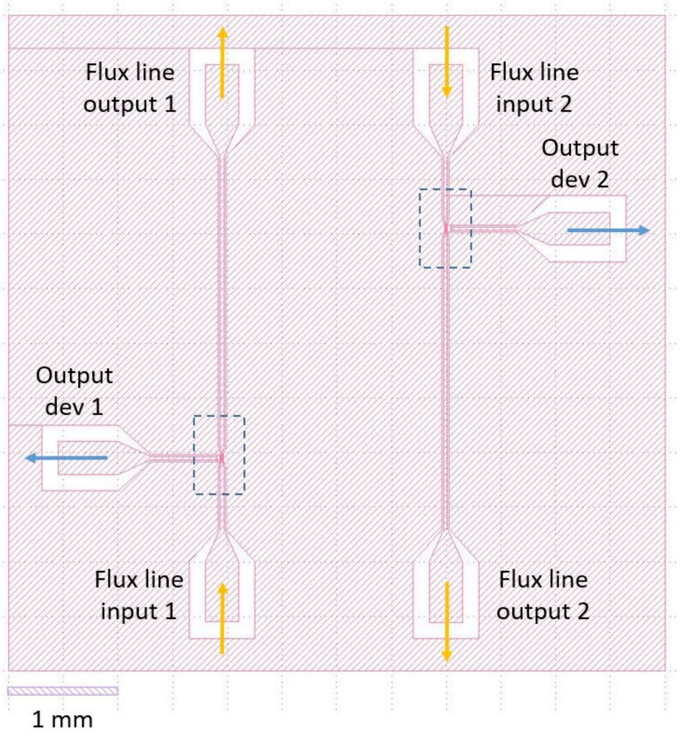

Supplementary Figure 7. CAD image of a typical chip with two identical frequency comb devices. The chip embeds two output lines connected to two identical SQUIDs, powered by two flux lines with input and output ports. The dased rectangles indicate the area where the zoom in Figure 7 is taken.

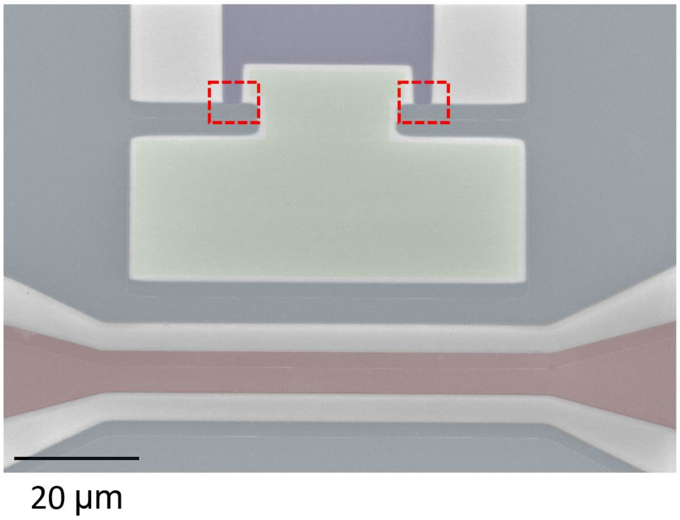

Supplementary Figure 8. SEM false color image of the SQUID plus flux line. In red one can see the signal line of the flux CPW, in purple the one of the output CPW, in blue the ground plane and in green the SQUID loop area. Finally, the Josephson junctions are highlighted in the red dashed squares.

SUPPLEMENTARY NOTE 6: DEVICES DESIGN

SUPPLEMENTARY NOTE 7: DERIVATION OF EQUATION 1

When two Josephson tunnel junctions are connected in parallel and embedded in a superconducting loop, the total Josephson current through the system is [4]

$$\begin{aligned} I_J &= I_{c1}\sin\varphi_1 + I_{c2}\sin\varphi_2 \\ &= (1+r)\sin\varphi_1 + (1-r)\sin\varphi_2, \end{aligned} \tag{S3}$$

where I_{cj} and φ_j represent the adimensional critical current and the phase drop of junction j, and r is the asymmetry parameter between the critical currents of Eq. 1 in the main text. The goniometric relation $\sin\varphi_1 + \sin\varphi_2 = 2\cos\frac{\varphi_1-\varphi_2}{2}\sin\frac{\varphi_1+\varphi_2}{2}$ has to coexist with the constraint between the phases due to the fluxoid quantization, $\varphi_2 - \varphi_1 = \frac{2\pi\Phi}{\Phi_0}$ when an external flux Φ is applied. The combination yields $\sin\varphi_1 + \sin\varphi_2 = 2\cos\phi\sin\varphi$, where $\phi \equiv \pi\Phi/\Phi_0$ and $\varphi \equiv (\varphi_2+\varphi_1)/2$.
Equation S3 can be rewritten as

$$I_J = \sin\varphi_1 + \sin\varphi_2 + r(\sin\varphi_1 - \sin\varphi_2). \tag{S4}$$

Since $-\sin\varphi_2 = \sin(-\varphi_2)$, with the substitutions above Eq. S4 becomes

$$\begin{aligned} I_J &= 2(\cos\phi\sin\varphi + r\sin\phi\cos\varphi) \\ &= (I_{c1}+I_{c2})(\cos\phi\sin\varphi + r\sin\phi\cos\varphi). \end{aligned} \tag{S5}$$

[1] B. I. Bleaney and B. Bleaney, Electricity and Magnetism, Volume 2, Vol. 2 (Oxford University Press, USA, 2013).
[2] J. A. Delport, K. Jackman, P. l. Roux, and C. J. Fourie, IEEE Transactions on Applied Superconductivity **29**, 1 (2019).
[3] R. Gross, A. Marx, and F. Deppe, Applied Superconductivity: Josephson Effect and Superconducting Electronics, De Gruyter Textbook Series (Walter De Gruyter Incorporated, 2016).
[4] J. Clarke and A. I. Braginski, The SQUID handbook (John Wiley & Sons, 2006).
